# Metal Pollution and Bioaccumulation in the Nhue-Day River Basin, Vietnam: Potential Ecological and Human Health Risks

**DOI:** 10.3390/ijerph182413425

**Published:** 2021-12-20

**Authors:** Huong Thi Thuy Ngo, Lan Anh Thi Tran, Dinh Quoc Nguyen, Tien Thi Hanh Nguyen, Thao Thanh Le, Yue Gao

**Affiliations:** 1 Faculty of Biotechnology, Chemistry and Environmental Engineering, Phenikaa University, Hanoi 12116, Vietnam; tien.nguyenthihanh@phenikaa-uni.edu.vn (T.T.H.N.); thao.lethanh@phenikaa-uni.edu.vn (T.T.L.); 2 Bioresource Center, Phenikaa University, Hanoi 12116, Vietnam; 3 Institute of Tropical Biology, Vietnam Academy of Sciences and Technology, Hochiminh City 71351, Vietnam; tranlananh.k13tns@gmail.com; 4 Economic Geology and Geomatics Department, Vietnam Institute of Geosciences and Mineral Resources, Hanoi 12109, Vietnam; dinhnq@gmail.com; 5 Analytical, Environmental and Geo-Chemistry, Vrije Universiteit Brussel, 1050 Brussels, Belgium; Yue.Gao@vub.be

**Keywords:** metal pollution, Nhue-Day River basin, freshwater fish, ecological risk assessments, health risk assessments

## Abstract

(1) Background: Metal pollution in the Nhue-Day River basin has impacted approximately 12 million people. However, none of the previous studies considered the entire basin’s environmental and health risks. Thus, this research aims to fill knowledge gaps and reduce risks. (2) Methods: Sediment and fish samples from the basin were analyzed to determine the levels of Zn, Cu, Pb, and Cd pollution and their potential ecological (EF, modified Pollution Index—mPI, and expanded, modified potential ecological risk index—emRI) and human health risks (THQ, HI, and TR indices). (3) Results: Metal levels in sediment exceeded Canadian aquatic life protection guidelines, indicating moderate to severe contamination (EFs: 1.3–58.5 and mPIs: 4–39). Compared to the new proposed ecological risk threshold, all river sites and Site 1 for ponds had elevated metal levels; and these posed a very high ecological risk in spring (emRI > 4.5), with Cd being the most hazardous. Lead levels in all fish tissues surpassed Vietnamese and EU food regulations. In agreement with THQ, EWI (Zn, Cu) and EMI (Cd) were both less than 2.5% of the PTWI and PTMI, respectively. However, HI values of 0.67–1.26 suggested a moderate health risk. Carcinogenic risk (TR > 10^−6^; estimated for Pb) was detected in several localities for Common carp and Tilapia during the warm season. (4) Conclusions: Metals had a negative impact on the basin’s ecosystem, with Cd being the most dangerous. Because of lead, consumption of Common carp and Tilapia from the basin may pose both non-carcinogenic and carcinogenic health concerns.

## 1. Introduction

Metal pollution in the aquatic ecosystem, including the river basin, is unavoidable due to rapid industrialization and urbanization [1]; and it has a negative impact on the health of the ecosystem and, hence, aquatic organisms such as fish. Furthermore, the agriculture and aquaculture sectors may be influenced because the polluted river water is used for crop irrigation and aquaculture ponds. Thus, consuming potentially contaminated products puts human health at risk [2,3].

In recent decades, under the pressure of economic development, Vietnam has entered a rapid industrialization and urbanization period, putting the environment at risk of extreme degradation. Metal contamination in water and sediments has become unavoidable and has been observed in several riverine ecosystems, including the Red River [4,5], the Nhue River [6], and the Dong Nai River [7,8]. The Nhue-Day River system, for example, has piqued the public’s interest due to its high contamination with organic materials and metals [4,5]. The Nhue and Day rivers in Vietnam are distributaries of the Red River, which flows through Hanoi and four provinces (Hoa Binh, Ha Nam, Ninh Binh, and Nam Dinh) before joining at Phu Ly City in Ha Nam province. This river basin (covering an area of about 7655 km^2^ and a length of 236 km) was once rich in biodiversity and vital to the economic development of about 12 million inhabitants. The rivers provide most of the freshwater for agriculture and aquaculture in the region. As a result of discharged effluents from large residential areas, such as Hanoi, which has over 4000 industrial establishments, nearly 500 traditional craft villages, and about 1400 hospitals and healthcare establishments, the water quality of these rivers has deteriorated [9]. Metal pollution in the basin can pose severe threats to natural ecosystems due to its toxicity, persistence, and bioaccumulation [10]. As a result, it may affect aquatic species such as fish [11] and humans who consume these organisms.

Metal-contaminated sediment and aquatic products have been extensively studied globally [12,13]. When river basin residents use polluted water for agriculture and aquaculture systems, food safety concerns have also been raised [14,15,16]. In recent years, there has been an increase in environmental-related diseases, and evidence of trace elements and their bioavailability influences animals’ health, such as cancer and autoimmunity [17,18]. Numerous other elements, including As, Cd, Hg, Mn, and Pb, are also neurotoxic, impairing areas of the brain involved in language, memory, and executive function, as well as psychosocial behavior, particularly in children [19]. As a result, more effort and money have been expended, attempting to prevent and control these diseases [20]. Long-term ingestion of trace metal-contaminated foods has been linked to poisoning, neurotoxicity, genomic instability, and cancer [19,21], particularly when trace metal uptake exceeds the recommended daily, weekly, or monthly intake levels. These metals are almost entirely derived from fish, a high protein and omega fatty acid source [22]. Fish is a popular part of the human diet in Vietnam and other parts of the world. However, regular consumption of metal-contaminated fish may pose a health risk to consumers. Thus, it is critical to assess the ecological risk of metals in sediment and the human health risk of metal exposure from foods [23], but such research is still scarce in Vietnam. They either covered small areas [6,15,16] or only addressed the risk of sediment-borne metal contamination [5,24]. None of the previous studies considered the entire river basin or the link between metals in sediment and their transfer through the food chain to pose a risk to humans. Therefore, we hypothesize that if the basin sediment is polluted with metals, the ecosystem will be at risk, as will human health from consuming fish containing ingested metals. In order to test these hypotheses, this study was conducted to investigate: (1) the pollution status of metals (Zn, Cu, Cd, and Pb) in the surface sediment on both temporal and spatial scales; (2) the potential ecological risks posed by these metals in the basin’s surface sediment; (3) metal bioaccumulation in three important freshwater fish, namely Common carp (*Cyprinus carpio*, Linnaeus, 1758), Silver carp (*Hypophthalmichthys molitrix*, Valenciennes 1844), and Tilapia (*Oreochromis niloticus*, Linnaeus, 1758); and (4) the potential human health risks of consuming these fish from the Nhue-Day river basin.

Because of their toxicity and popularity in the basin, these metals were chosen for this study. The potential effects of metals on fisheries, a significant economic sector in Vietnam (3.43 percent of national GDP in 2018), and their repercussions on human health and the environment must be urgently addressed. This study aims to help close these knowledge gaps and create a database of potential risks to Vietnam’s ecosystem and human health, allowing the government to make timely decisions and plan for long-term basin management. It also contributes to lowering the risk of consuming fishery and aquaculture products in Vietnam and elsewhere.

## 2. Materials and Methods

### 2.1. Study Sites and Samples

**Figure 1:**
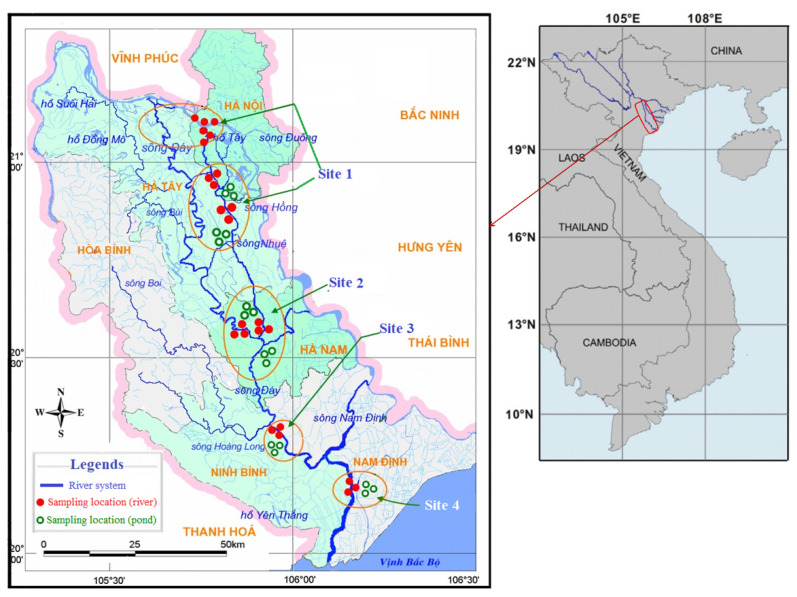
A map of the Nhue-Day River basin with sampling locations at four study sites. Closed red dots indicate sampling locations in rivers, while open green dots illustrate locations in aquaculture ponds.

Most aquaculture ponds in the basin use polluted water from the Nhue-Day River system. Therefore, to test the hypotheses and to assess the effects of river water on aquaculture ponds, 36–42 sediment and 24–40 fish samples from each season were collected from the rivers (24 locations) and ponds (18 locations) at four study sites within the Nhue-Day River basin (Figure 1). The sampling areas include the Nhue river from Hanoi Capital (Site 1, at the confluence with the Red River) to Ha Nam Province (Site 2, at the confluence with the Day River), as well as the downstream of the Day River in Ninh Binh Province (Site 3, at the confluence with the Hoang Long River) and Nam Dinh Province (Site 4, after the confluence with the Nam Dinh River). This region is in the northern plains at 20°–21°20′ N and 105°–106°30′ E, and it has a subtropical monsoon climate with four seasons, including a dry-cold winter and a rainy-hot summer. A total of three fish species commonly raised in this basin and which are essential food sources for people in northern Vietnam [15] were chosen. *C. carpio* is a European and Asian benthopelagic fish. The herbivorous filter feeder *H. molitrix* is native to North and Northeast Asia. *O. niloticus* is an omnivorous benthopelagic fish native to Africa. Samples were collected in four seasons between 2013 and 2014: spring (April), summer (July), autumn (September), and winter (December). Other data on aquaculture status and fish consumption in the river basin were collected between 2015 and 2017.

### 2.2. Chemicals, Labware, and Sample Collection

All the chemicals used in the metal analysis were of the analytical grade (Merck, Darmstadt, Germany). Analytical containers and tools were cleaned with tap water, soaked in 1:1 conc. HNO_3_ for 24 h, rinsed with double-distilled water and dried before use.

Triplicate sub-samples of sediment were collected at a depth of about 0–20 cm in a triangle using a Petersen grab (Cole-Parmer, Hanoi, Vietnam), mixed well, kept in a double-zipped polyethylene bag, and transported cold to the laboratory. Marketable-sized fish were caught in ponds (cultivated fish) or purchased from local fishermen (wild fish) and transported alive to the laboratory for dissection.

### 2.3. Metal Analysis

The collected sediments were dried in an oven at 70 °C for three days, ground with a mortar and pestle, sieved through a 63-µm sieve (No. 230), and digested as described by Le and Ngo [25] with some modifications. Briefly, in borosilicate glass tubes, an aliquot of 100–150 mg of ground sediment was digested with a mixture of 5 mL of suprapur HNO_3_ (65%, *v*/*v*), 2.5 mL of HF (40%, *v*/*v*), and 1 mL of HCl (30%, *v*/*v*) (room temperature for 24 h, then added 0.5 mL of H_2_O_2_ and digested at 120 °C for at least 10 h). After cooling to room temperature, samples were diluted to 20 mL with bidistilled water and filtered through a 0.45-µm cellulose membrane syringe filter (Whatman, Singapore). Total Fe in the sediment was determined by using Phenanthroline Method [26].

The fish were anesthetized before being dissected for muscle, gill, liver, and kidney tissues. About 100–200 mg wet weight (ww) of each tissue was transferred to a Teflon tube for wet digestion [27] in a digestion box (bio-carrier, Nagel, Bremen, Germany) with 2.5 mL of aqua regia (conc HNO_3_ and conc HCl, *v/v* 1:3) and 200-µL of 30% H_2_O_2_ at 120 °C for at least 5 h. The samples were then diluted to 20 mL with bidistilled water and filtered through a 0.45-µm cellulose membrane syringe filter. ICP-MS was used to analyze all sediment and fish tissue samples after filtration (Varian Ultra Mass 700, Victoria, Australia).

The analytical procedure’s accuracy was tested and validated by analyzing certified reference materials SRM688 (Basalt rock) and DOLT-4 (Dogfish liver). Every batch of samples contained a procedural blank and a certified material sample, which were treated and analyzed in the same manner as the samples. The metals’ ICP-MS working standards were made from stock solutions of their nitrate salts in HNO_3_ (0.5 mol/L) by dilution with appropriate volumes of 2% HNO_3_. Zn, Cu, Pb, and Cd recoveries ranged from 84 to 120% for SRM688 and 87 to 113% for DOLT-4. Cu and Zn had detection limits of 1 µg/L, while Cd and Pb had detection limits of 0.001 µg/L. All metal concentrations in fish samples were calculated on a wet weight basis (mg/kg ww) and in sediment samples on a dry weight basis (mg/kg dw).

### 2.4. Risk Assessments

#### 2.4.1. Ecological Risk Indices

The single element index, enrichment factor (EF) [28], was used to assess the pollution level of a specific metal (Equation (1)).

(1) EF=(CiCref)Sample(CiCref)background

C_i_ denotes the concentration of a given element, and C_ref_ denotes the concentration of a normalization element; in this case, Fe was used because it is so abundant that anthropogenic sources have little effect on its natural variations in the sediment. Qingjie et al. [28] proposed seven contamination levels: EF < 1, unpolluted or no polluted; 1 < EF < 3, slightly polluted; 3 < EF < 5, moderate polluted; 5 < EF < 10, from moderately polluted to strongly polluted; 10 < EF < 25, strongly polluted; 25 < EF < 50, from strongly polluted to extremely polluted; and Ef > 50, extremely polluted. An EF > 1 indicates anthropogenic enrichment of an element [29].

The modified Nemerow Pollution Index (mPI) [30,31] and the expanded, modified potential ecological risk index (emRI) [32] were used to assess sediment quality and potential ecological risks, considering the variation in toxicity of each metal and the effects of the element mixture in sediment, particularly considering the complex sediment behavior.

(2) $$\mathrm{mPI}=\sqrt{\frac{\begin{array}{cc} \begin{array}{cc} {\left({\mathrm{EF}}^{\mathrm{mean}}\right)}^{2} & + \end{array} & {\left({\mathrm{EF}}^{\max}\right)}^{2} \end{array}}{2}}\;$$

(3) $$\mathrm{emRI}=\frac{\sum_{i=1}^{n}{\mathrm{mE}}_{r}^{i}}{\sum_{i=1}^{n}T_{r}^{i}}=\frac{\sum_{i=1}^{n}\begin{array}{ccc} T_{r}^{i} & \times & {\mathrm{EF}}^{i} \end{array}}{\sum_{i=1}^{n}T_{r}^{i}}\;$$

According to Brady et al. [31], there are six mPI classifications: mPI < 1, unpolluted; 1 < mPI < 2, slightly polluted; 2 < mPI < 3, moderately polluted; 3 < mPI < 5, moderately-heavily polluted; 5 < mPI < 10, heavily polluted; and 10 < mPI, severely polluted.

Hakanson [33] introduced the term RI, which was later modified by Duodu et al. [34] to become mRI, which was calculated using EF rather than contamination factor (Cf) to minimize the limitation of overestimation of contamination level. The RI was originally proposed to estimate the potential ecological risk of all eight contaminants: Hg, Cd, As, Pb, Cu, Cr, Zn, and PCB. However, the composition of metals in sediment varies by ecosystem, depending on environmental conditions and pollution sources. Therefore, emRI is more appropriate for estimating the potential ecological risk of a given ecosystem with its specific pollution pattern [32]; Er denotes a nominal potential ecological risk factor; Tr denotes a dimensionless toxic factor for a specific element: Cd = 30, Pb = Cu = 5, and Zn = 1. Because data for the background level of metals in this river basin and region are unavailable, the upper continental crust values [35] were used as the background in this study (Cd = 0.098, Cu = 25, Pb = 20, Zn = 71 μg/g dw).

#### 2.4.2. Human Health Risk Indices

The following parameters [36] were calculated for the human health risk assessment of metal intake from fish consumption: the estimated daily intake (EDI), target hazard quotient (THQ), target cancer risk (TR), and total hazard index (HI).

(1) The EDI was calculated to determine the potential health risk posed by a particular metal:
  EDI = (Mc × IR)/Bw(4)
  where EDI is the estimated daily intake of a given metal from fish consumption for the local population (μg/kg bw/day); Mc is the metal concentration in fish (μg/g ww); IR is the daily fish ingestion rate (g/person/day), and Bw is the average body weight (kg). According to the FAO Food Balance Sheet for 2018, the average freshwater fish consumption by Vietnamese people was around 41 g/day/capita (http://www.fao.org/faostat/en/#data/FBS, accessed on 23 June 2021), and these data were used to calculate the IR of the young women group between the ages of 18 and 25, who have the lowest weight (51 kg, according to current survey data) and are thus more sensitive to metal exposure [15]. The estimated weekly intakes (EWIs; μg/kg bw/week) for Zn and Cu, as well as the estimated monthly intakes (EMI; μg/kg bw/month) for Cd (due to the exceptionally long half-life of cadmium), were calculated based on EDI and compared to the prescribed Provisional tolerance weekly intakes (PTWIs; μg/kg bw/week). The Joint FAO/WHO Expert Committee on Contaminants in Food [37] established these standards, with Zn and Cu at 7000 and 3500 μg/kg bw/week, respectively, and Cd at 25 μg/kg bw/month. For each metal, the acceptable daily intake (ADI; μg/kg bw/day) was calculated to compare with EDIs. FAO/WHO withdrew the PTWI for Pb in 2010; as a result, no comparison with this element was made.
(2) The non-carcinogenic (THQ) and carcinogenic target risks (TR) were calculated using the US EPA Regional screening level (RSL) and the Integrated risk information system (IRIS) for 2021, following the US EPA integrated risk analysis [38].
  (5) $$\mathrm{THQ}=\frac{\;\left(\mathrm{Mc}\;\times \;\mathrm{IR}\;\times 10^{-3}\times \;\mathrm{Ef}\;\times \;\mathrm{Ed}\right)}{\left(\mathrm{RfD}\;\times \;\mathrm{Bw}\;\times \;\mathrm{ATn}\right)}$$
  (6) $$\mathrm{TR}=\frac{\;\left(\mathrm{Mc}\;\times \;\mathrm{IR}\;\times 10^{-3}\times \;\mathrm{CPSo}\;\times \;\mathrm{Ef}\;\times \;\mathrm{Ed}\right)}{\left(\mathrm{Bw}\;\times \;\mathrm{ATc}\right)}$$
  where:
  Mc denotes the metal concentration in fish muscles (μg/g ww).
  Ef is the frequency of exposure (assuming 365 days per year).
  Ed is the total lifetime exposure duration (70 years).
  RfD is the oral reference dose of each metal (μg/g/day for Cu, 0.3 for Zn, and 0.001 for Cd [39].
  Bw is the average female body weight (51 kg).
  ATn and ATc are the average exposure times for non-carcinogens and carcinogens, respectively (365 days/year x exposure years, assuming 70 years).
  CPSo is the oral carcinogenic potency slope (risk per μg/g/day; CPSo of Pb 8.5 × 10^−3^).

It was assumed that cooking did not affect the concentration and toxicity of metals in fish [40]. The amount of metal absorbed from other food and drink sources was not considered. There is no RfD for Pb because there is no evidence of a threshold below which a non-harmful intake could be permitted [37]. As a result, the THQ for Pb was calculated as follows [41]:

THQ = Mc/MRL(7)

where MRL is the maximum residue level set by the European Commission (MRL for Pb is 0.3 μg/g ww) [42].

The total effects of multiple metals in fish (HI) were calculated, which is a sum of all calculated THQ values for all metals in each fish.

(8) $$\mathrm{HI}=\sum_{i=0}^{n}{\mathrm{THQ}}^{i}$$

When THQ or HI is lower than 1, there is no, or little health risk associated with consuming a specific fish in the basin. When the THQ or HI is higher than 1, and TR levels are higher than 1 × 10^−6^, an adverse health effect [38] and cancer risk [43], respectively, may be posed to fish consumers.

### 2.5. Data Processing and Analysis

All data collected were processed and analyzed using Microsoft Excel. To detect differences in metal concentrations in sediment and fish across four sites and four sampling seasons, a two-way analysis of variance (ANOVA) was used. If statistically significant differences (*p* < 0.05) were found, the Tukey–Kramer multiple comparisons test was applied (GraphPad InStat, San Diego, CA, USA).

## 3. Results and Discussion

### 3.1. Metal Pollution in the Nhue-Day River Basin: Spatial and Temporal Variation

**Figure 2:**
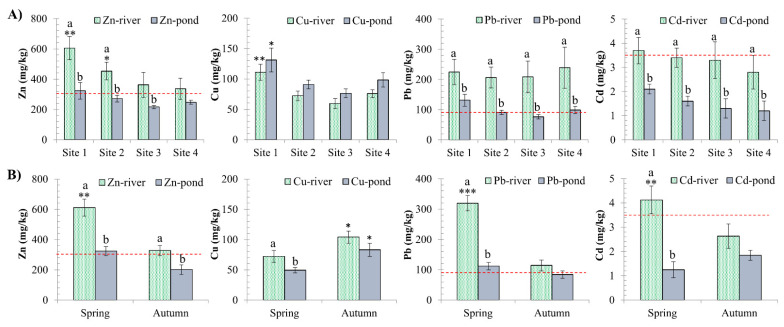
Spatial (**A**) and seasonal variations (**B**) of metals in sediment (mg/kg). Comparison with the Vietnamese standard Guideline for protecting aquatic life [39] (red dashed line). Significant differences between sampling sites (**A**) and times (**B**) in comparison to the lowest value (*) are indicated (mean ± SE, n = 10–20, * *p* < 0.05, ** *p* < 0.01, *** *p* < 0.001). Different letters (a > b) indicate that values from the ponds and the rivers at the same locations (**A**) or at the same times (**B**) are significantly different (*p* < 0.05; ANOVA followed by Tukey–Kramer multiple comparisons test).

The spatial and temporal distributions of metals in river and pond sediments were investigated (Figure 2). A two-factor ANOVA revealed statistically significant (*p* < 0.05) differences between sampling sites and seasons.

In terms of spatial variation, Zn and Cu in the rivers were higher at Site 1 (*p* < 0.05) and decreased towards Sites 2 and 3. (Figure 2A). All investigated metals tend to be higher in the pond at Site 1 (Hanoi), with Cu, Cd, and Pb being significantly higher than those at other sites (*p* < 0.05; Figure 2A). Surprisingly, almost all metals had higher values upstream. An explanation for this could be that the Red River section at Site 1 receives industrial effluents from the three industrial zones that are only a few km away from the river, namely Thang Long, Quang Minh, and Noi Bai. Nguyen et al. [5] discovered high levels of metals in the sediment of several Red River upstream sections. Metal pollution in this upper site may also be caused by industrial parks and traditional craft villages around Hanoi (Site 1).

Significant seasonal differences in Zn, Cd, and Pb levels in surface sediment have been observed: higher levels of Zn, Cd, and Pb in the river during spring (*p* < 0.01), but not in the pond (*p* > 0.05; Figure 2B) compared to autumn. Cu was relatively stable throughout the year, both in the river and pond (*p* > 0.05; Figure 2B). The sediment metals were about two times higher in the river than in the ponds, particularly Cd and Pb, and were ranked as Zn > Pb > Cu > Cd (Figure 2). Water showed a similar pattern, albeit to a lesser extent (data not shown). Because the water in aquaculture ponds is drawn from rivers, the metal levels in the pond fluctuate in accordance with the river water, particularly in the case of Cd and Pb. Metal accumulation in river sediment reflects water accumulation over time, but not in pond sediment.

In comparison to the sediment quality guidelines (SQG), the concentrations of three metals (Zn, Pb, and Cd) were higher than the Vietnam national standards for the protection of aquatic life [44] at almost all river locations (Table 1), particularly during the spring (Figure 2). When compared to the Canadian guidelines for the protection of aquatic life [45], all tested metals in sediment from all sites exceeded the Canadian standards by two to seven times (Table 1). In this basin, which is frequently inundated with contaminated water from industrial parks, traditional craft villages, and Hanoi City, high metal levels in the sediment result from sedimentation and retention of the metals from the water column [46]. Metals in sediment may be released back into the water column as environmental conditions change, such as temperature, pH, redox potential, oxygen concentration, and organic matter degradation [47], making sediment a potential secondary source of pollutants [48]. Consequently, high metal levels in sediment can pose a significant risk to the health of the aquatic ecosystem.

### 3.2. Metals Bioaccumulation in Fish

**Figure 3:**
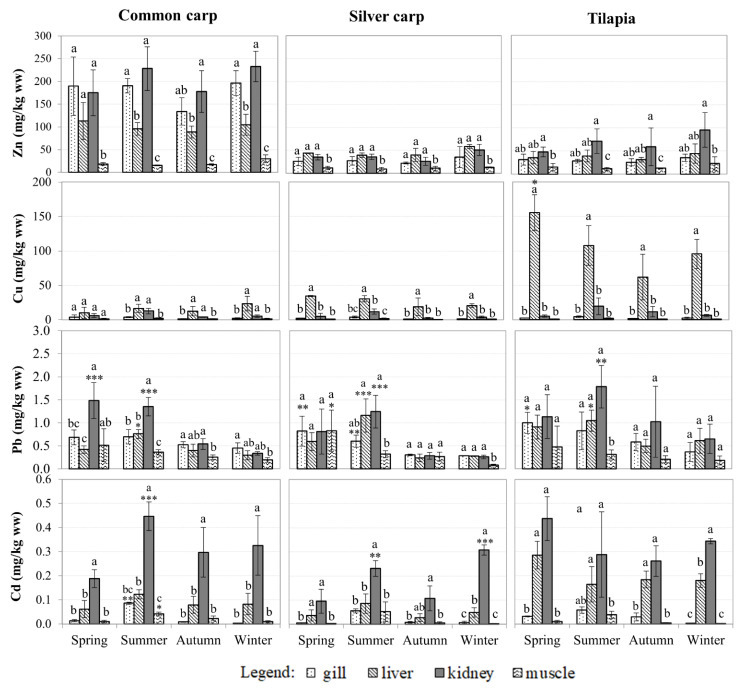
Concentrations of Zn, Cu, Pb, and Cd in various fish tissues (mg/kg ww) collected during different seasons. Asterisks (*) indicate statistically significant differences between seasons when compared to the lowest value in each organ (mean ± SE, n = 5–20, * *p* < 0.05, ** *p* < 0.01, *** *p* < 0.001). Different letters (a > b > c) denote statistically significant differences in metal levels between organs in each season (*p* < 0.05; ANOVA followed by Tukey–Kramer test).

Fish were mainly collected from ponds (>85%) because no fish were found at Site 1, and they were scarce at other sites. As a result, unlike sediment samples, fish were not classified into pond and river groups. The metal levels in the tissue may also be affected by the weight of the fish. Therefore, fish of similar sizes were collected for this study. Metal bioaccumulation is most commonly found in the kidney and liver (*p* < 0.05), followed by the gills and muscles (Figure 3). Higher metal levels in the liver indicate that the liver is the primary target organ of contaminants taken up via the intestine and the center for metal metabolism [49]. When chemicals are taken up through the gills, they also find their way to the kidney [49].

Zn accumulated at the highest concentrations in fish tissues (11–282 mg/kg ww), followed by Cu (0.6–267 mg/kg ww), Pb (0.09–2.7 mg/kg ww), and Cd (0.001–0.54 mg/kg ww) (Figure 3). Although the concentrations of Pb in sediment were higher than those of Cu (Figure 2), Cu accumulated at higher levels in fish tissues, indicating that different metals accumulate in fish via different pathways. Furthermore, because Zn and Cu are essential metals, they are accumulated at a higher level and are regulated by fish [50]. On the other hand, Cd and Pb are non-essential elements, and, therefore, tend to accumulate in organisms [51]. None-parametric Spearman’s Rank Correlation test revealed tight relationships between metals in sediment and those accumulated in fish tissues, specifically for Common carp: Zn, Cu, and Pb (*p* < 0.05), and for Tilapia: Zn, Cu, and Cd (*p* < 0.05). Thus, metals in the environment are linked to bioaccumulation and transfer through the food chain.

In comparison to Vietnamese [52] and international food safety standards [42,53] (Table 2), Cd levels in all liver and kidney samples, as well as some muscle samples, exceeded food safety standards; Zn levels also exceeded food safety standards in Common carp gills, liver, and kidney, and Tilapia kidney. Among the metals tested, lead posed the highest risk to fish consumers because its concentration in all four tissues surpassed the standards [42,52] (Figure 3; Table 2), except for in muscle samples collected in winter (Pb < 0.3 mg/kg ww). In this study, all metals found in fish muscle were significantly lower than the WHO’s [53] concentration threshold (Table 2).

Silver carp bioaccumulated metals at a lower level than other species in the liver, kidney, and gill (Figure 3), but not in muscle tissues because they live in the upper layer of the water column and feed on phytoplankton, and thus are less influenced by metals in sediment. Common carp and Tilapia are bottom-dwellers that feed on zooplankton and detritus. Therefore, they have been impacted by metal-contaminated sediment [54].

### 3.3. Risk Assessments

#### 3.3.1. Potential Ecological Risk of Metals in Sediment

**Figure 4:**
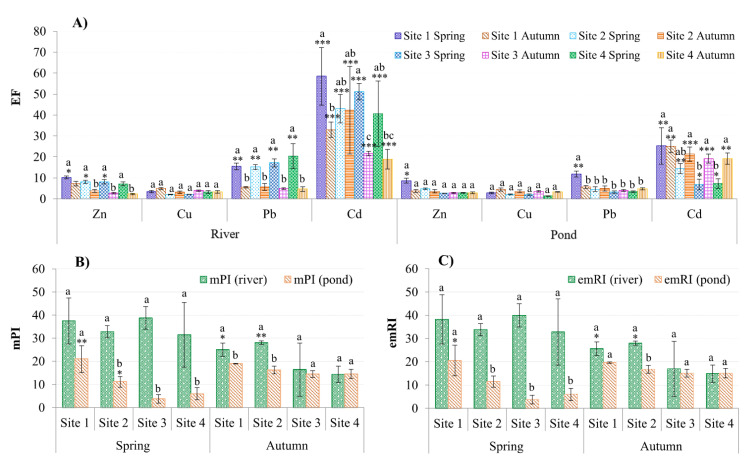
Ecological risk assessment for (**A**) single (enrichment factors) and (**B**,**C**) mixture of metals (mPI: modified Nemerow Pollution Index, and emRI: expanded, modified potential ecological risk index) in surface sediment from the Nhue-Day River basin. Asterisks (*) represent statistically significant differences between sites in each ecosystem (**A**) or in each season (**B**,**C**) when compared to the lowest value (mean ± SE, n = 3–12, * *p* < 0.05, ** *p* < 0.01, *** *p* < 0.001). Different letters (a > b > c) denote statistically significant differences between the site-season of each element (**A**) and between the ecosystems of each site (**B**,**C**) (*p* < 0.05; ANOVA followed by the Tukey–Kramer test).

According to the SQG, the concentrations of metals in the basin sediment exceeded the Canadian standard, as stated in Section 3.1 [45]. However, the SQG does not specify the sources of pollutants in the environment. Therefore, EF was used to assess sediment contamination (Equation (1)), with Fe as a normalization element with the least negligible influence from an anthropogenic source. All tested metals had EF values higher than one at all sites, and this could be due to the increased number of new industrial parks and traditional craft villages in the basin. EF values were significantly higher in the river than in the ponds, particularly for Cd and Pb (Figure 4A), and this is consistent with higher mPI and emRI values in the river sediment vs. ponds (Figure 4B,C). The findings revealed that rivers are the primary sinks for metals emitted by nearby industrial or manufacturing activities. The spatial differences are unclear, but the remarkably high EFs at all sites show that both rivers and ponds were moderate to severely polluted with high anthropogenic impacts, particularly Cd (EF ranged from 19 to 58 for rivers and 7–25 for ponds; Figure 4A).

Still, both the SQG and EF approaches focus on a single metal, whereas sediment toxicity to organisms is commonly caused by exposure to a mixture of elements. Therefore, the mPI and emRI (Equations (2) and (3)) were used to assess the levels of pollution and ecotoxicological risks of a mixture of tested metals in surface sediment. Calculated mPI values were compared to six categories of pollution levels from Brady et al. [31], and the results showed that the sediment in the basin ranged from moderately heavily polluted (3 < mPI < 5) to severely polluted (mPI > 10; Figure 4B) with metals. All mPI values were greater than four, with Ha Nam having the highest value of 28 (Site 2). The difference in pollution levels between spring and autumn is more pronounced in rivers (*p* < 0.05) than in ponds, but the differences between sites were apparent for samples collected from ponds in spring and rivers in autumn (Figure 4B).

Similarly, Hong et al. [32] proposed the emRI index to improve the method for determining the potential ecological risk, which can be applied to any condition (contaminant combinations and available testing methods). However, only using the emRI index does not allow us to categorize the risk level of a given ecosystem, which is a significant limitation, so the calculated result(s) need to be compared with the thresholds to determine the current risk level of the tested ecosystem. As those thresholds are not yet available for use with emRI, we combined the findings from Hakanson [33] and Hong et al. [32] to calculate them as given in Table 3, which clearly shows that the emRI ranges correspond to the ecological risk levels of the ecosystem, and provide a more accurate method. These new ecological risk thresholds for emRI were calculated as the relative values of the potential ecological risk index per toxic unit of the target toxic substance mixture, rather than as a sum of the potential ecological risk factors for all tested contaminants as was calculated for RI [33]. The calculation was based on Hakanson’s categories and the toxic factors of the eight contaminants used in the previous study (Hg, Cd, As, Pb, Cu, Cr, Zn, and PCB) [33]. As a result, these thresholds enable and facilitate the classification of potential ecological risks in various ecosystems of concern. Furthermore, these new thresholds can help avoid overestimation or underestimation of potential ecological risk indices, remarkably when the number of toxic substances included in the studies differs from Hakanson’s study [33]. As a practical example, only four metals (Zn, Cu, Cd, and Pb) were examined in this study; although As and Hg are more toxic and occur in this basin, they were omitted due to a limited budget and insufficient infrastructure to analyze. Thus, to overcome the limitation of using the sum of Er, we used emRI rather than RI to assess the ecological risk level of sediment in the basin.

Based on the calculated mEr, Cd posed a very high ecological risk at all sites (mEr > 320) in the rivers and ponds; Pb showed a moderate ecological risk at all river sites but not in the ponds (Table 4). However, the mEr calculated for Cu and Zn was lower than 40 at all sampling sites, indicating a low ecological risk. Cd posed a very high risk to the environment at every site in the spring, but Pb posed only a moderate risk in the river (*p* < 0.05). In ponds, no seasonal variation was observed (*p* > 0.05).

In accordance with the mPI results, the emRI for metals in sediment demonstrated a very high ecological risk (emRI > 4.5; Table 3) at all sites (Table 4), particularly at Sites 1 and 2 for rivers in autumn and ponds in spring (*p* < 0.05; Figure 4C). When emRI was compared between seasons, it was observed that metals in the spring have a higher ecotoxicological risk in rivers but not in ponds (*p* < 0.03; Figure 4C; Table 4). The emRIs in this study were approximately ten times higher than those found in the Tamsui River in northern Taiwan, with less than 2.3 at all studied sites [32]. Cd was the most significant contributor to the river sediment’s potential ecological risk, which may adversely affect the surrounding riverine ecosystems, including aquaculture ponds.

Because residents rarely eat fish livers, kidneys, or gills, the EDI was calculated solely on fish muscle (Equation (4)). EDI values for metals accumulated in the fish ranged from 0.006 to 20.9 μg/kg bw/day, in decreasing order of Zn > Cu > Pb > Cd (Table 5). The PTWI for Pb was withdrawn in 2010 after the Panel on Contaminants in the Food Chain (CONTAM) determined that the previously used PTWI for Pb (25 g/week/kg bw) was insufficiently protective [36]. As a result, the comparison can only be made for Zn, Cu, and Cd, and all EDIs were significantly lower than ADI standards (Table 5). A similar pattern can be seen when EWI is compared to PTWI (for Zn, Cu) or EMI is compared to PTMI (for Cd). Even though Cd posed the most significant ecological risk to the riverine ecosystem, Zn, Cu, and Cd levels in tested fish muscles pose little or no health risk to fish consumers. These findings are consistent with those of Marcussen et al. [15], who investigated Cd, Pb, and As levels in the muscles of Common carp, Silver carp, and Tilapia caught in Hanoi’s peri-urban areas. At Site 1 (Hanoi), residents appear to be exposed to higher metal concentrations when eating fish from this region, particularly Common carp. In summary, according to the EDIs, EWI, or EMI data, the average consumption of these fish accounted for only 0.11–2.4% of allowable intake (Table 5), posing no human health risk due to Zn, Cu, or Cd exposure. The amount of edible tissue consumed per month to reach the PTMI was high, averaging around 32 kg/month for Common carp, 49 kg/month for Tilapia, and 52 kg/month for Silver carp. It should be noted that this does not apply to other metals ingested by humans from fish and other foods.

#### 3.3.2. Human Health Risk Indices

**Figure 5:**
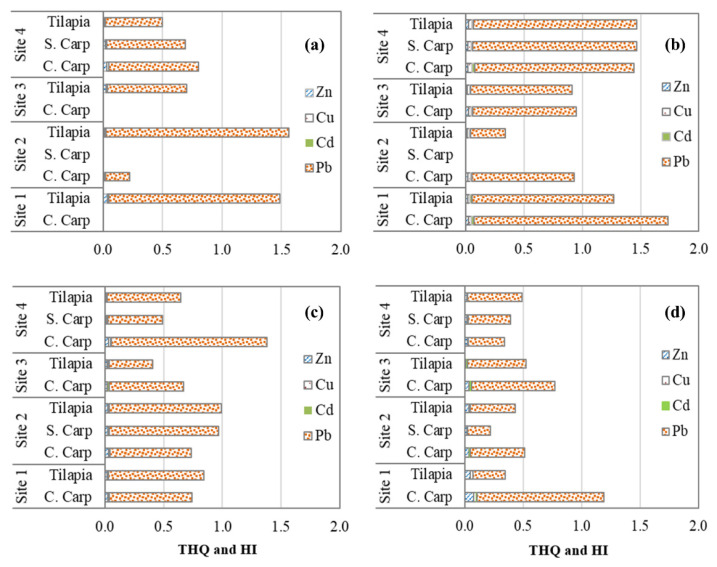
Seasonal and spatial target hazard quotient (THQ) and total hazard index (HI) of four metals in fish collected from four research sites: (**a**) Spring, (**b**) Summer, (**c**) Autumn, and (**d**) Winter.

Due to the fact that Pb could not be included in the risk assessment using EDI, non-carcinogenic (THQ, Equation (5)) and carcinogenic risks (TR, Equation (6)) were evaluated to gain a better understanding of the potential health risk to fish consumers. The THQs of four metals ranged from 0.0008 to 1.66, with THQ values of Zn, Cu, and Cd less than one in all investigated fish and at all sites (Figure 5), indicating no potential health risk for consumers. These results are consistent with the EDI, EWI, and EMI results discussed previously. However, THQ values for Pb exceeded one in several locations at Sites 1 and 4, with an average value of approximately 18, 29, and 64 times that of Zn, Cu, and Cd, respectively, indicating a potential health risk to fish consumers. Among species, Common carp had the highest THQs, followed by Tilapia and Silver carp (Figure 5). The average THQs were as follows in terms of sampling periods: summer (0.31) > spring (0.27) > autumn (0.20) > winter (0.14). This varied across sites, decreasing in order of Site 1 (0.29) > Site 4 (0.22) > Site 2 (0.19) ≥ Site 3 (0.186).

**Figure 6:**
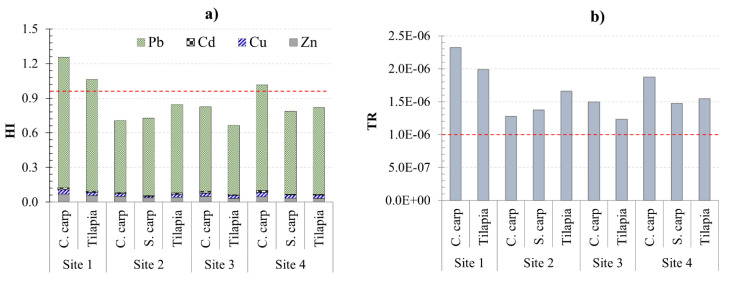
Year-average values for (**a**) the target hazard quotient (THQ) and the total hazard index (HI) of four tested metals; and (**b**) the target cancer risk (TR) posed by Pb in three fish species from four research sites. HI < 1 and TR < 1 × 10^−6^, acceptable risk; HI > 1 and 1 × 10^−6^ < TR < 1 × 10^−4^, potential risk.

HI was calculated by considering the total effects of four metals in fish (Equation (8)). The year’s average HI values ranged from 0.67 to 1.26, with both Common carp and Tilapia exceeding one (Site 1; Figure 6a), indicating a potential health risk for regular fish consumption. When all sampling seasons and locations are considered, the potential health risk may occasionally appear at Sites 1 and 4, particularly during the summer (Figure 5). Pb was the major contributor to the total health risk among the metals studied, with its THQ values accounting for between 88 and 92% of HI. The current findings were consistent with those of Wang et al. [13], who discovered THQ > 1 in only one of eight coastal provinces. In contrast, Tao et al. [55] and Taweel et al. [56] found lower HI values (<1) of these metals in eight freshwater fish species from Taihu Lake in China and Tilapia from two lakes in Malaysia, respectively. This contradiction may be that the studies used variables such as consumption rate and average body weight, which may be country-specific.

TR values for Pb ranged from 4.0 × 10^−7^ to 3.4 × 10^−6^, indicating a potential carcinogenic risk to fish consumers in some areas, particularly Hanoi and Nam Dinh (Sites 1 and 4; Figure 6b). These high TRs coincide with the high HI values at Site 1 and, to a lesser extent, Site 4 (Figure 6a). When sampling locations and seasons were considered, TRs produced similar results to HI, i.e., a high possibility of carcinogenic health risk was found at Sites 1 and 4, particularly in summer samples (data not shown). These findings raise the question of fish culture and crop irrigation using water from these rivers, particularly in the Hanoi region (Site 1). In summary, Cd is the primary contributor to ecological risk, but Pb is the primary contributor to human health risk due to the new THQ calculation method applied to this element.

The authors are responsible for setting parameters for using THQ, HI, and TR values in risk assessment studies, depending on the purpose of the study, region, and country. Thus, HI and TR values should be used to aid decision-making. This study found HI values higher than one in some locations and TR values greater than 1 × 10^−6^ in the most studied sites, implying the possibility of non-carcinogenic and carcinogenic effects on human health, respectively, when consuming wild and cultured fish from the basin.

In addition to the four metals investigated in this study, other toxic pollutants also impact the fish and environment of the Nhue-Day River basin. Furthermore, because it is a low-cost source of both water and nutrients, untreated wastewater is commonly used as a primary water source for agriculture and aquaculture. Those sources of toxic substances are transported through the food chain and additionally contribute to human health risks. Water spinach grown in Hanoi posed a low health risk for human consumption [16], but high Pb intakes from water spinach were recorded in 14.1% of the surveyed residents in Ha Nam [6]. THQs and HIs are also commonly linked to a subset of pollutants, so adding more pollutants to the study may increase HI values. Moreover, the HI and TR equations ignore the potential mutual effects of various pollutants or the effects of other environmental factors on metal toxicity. Therefore, future research should be conducted in the Nhue-Day River basin to assess potential health risks related to contaminated foods, including a broader range of toxic substances and environmental factors.

## 4. Conclusions

Metal concentrations in the sediment of the Nhue-Day River basin varied over space and time (*p* < 0.05) and were highest at Site 1 (vs. other sites), as well as during spring (vs. autumn) for Cd, Zn, and Pb. Metal concentrations in sediment were higher than the Canadian standard (CCME, 2007) for the protection of aquatic life at all study sites. The EF values at all sites are remarkably high, indicating that both rivers and ponds are moderate to severely contaminated with anthropogenic metals, particularly Cd (EF ranged from 19 to 58 for rivers or from 7 to 25 for ponds). The higher EF and mPI found in rivers indicate that rivers are the primary metal sinks. All calculated mPIs ranged from 4 to 39, indicating that the basin’s sediment was moderate to severely polluted. Cd was found to pose the highest potential ecological risk at all sites (mEr > 320), followed by Pb (40 mEr 80) at river sites, and Cu and Zn posed a low ecological risk. In comparison to the new ecological risk thresholds (Table 3), emRI revealed a high risk of the metal mixture (emRI > 4.5), particularly in the rivers during spring. Cd is the most dangerous element in river sediment and may harm the surrounding ecosystem.

For common carp (Zn, Cu, and Pb) and Tilapia (Zn, Cu, and Cd), there were clear (*p* < 0.05) links between metals in the sediment and those accumulated in fish muscle. Metal accumulation was higher in Common carp and Tilapia than in Silver carp, with the kidney and liver being the primary sites. Pb levels in fish muscle tissues had the highest threat to consumers, followed by Cd, because they exceeded national and international food standards [42,52]. However, all calculated values of EDI, EWI (for Zn, Cu) or EMI (for Cd) for muscles are well below the FAO/WHO recommendations (ADI, PTWI, and PTMI; JECFA, 2011), indicating that there is no risk of Zn, Cu, or Cd for people consuming fish from the basin.

The results showed that consuming fish (especially Common carp, followed by Tilapia) from some regions (Sites 1 and 4) poses a human health risk for Pb (THQs > 1) but not for the other three metals (THQs < 1). In the summer, HI (0.67–1.26) showed an overall potential health risk for regular fish consumers only at Site 1, with occasional appearances at Site 4. Pb contributed the most to total risk among metals, with THQ values accounting for 88 to 92% of HI. Furthermore, carcinogenic risk (TR > 1 × 10^−6^, calculated for Pb) to fish consumers was observed in almost all regions, particularly with Common carp and Tilapia. Thus, Cd in sediment poses the greatest threat to the basin’s ecology, Pb in Common carp and Tilapia poses the greatest threat to human health. The findings of this study will be helpful for long-term basin management and, ultimately, for river basin and public health management.

## Figures and Tables

**Table 1:** Metal concentrations in sediment from rivers and ponds (mean ± standard error of mean) in the current study compared to Vietnamese and Canadian guideline levels (mg/kg).

Metals		Concentration (mg/kg; Present Study)				^i^ Guidelines	
		Site 1	Site 2	Site 3	Site 4		
Cd	River	4.2 ± 1.0	4.4 ± 1.1	3.1 ± 0.64	2.8 ± 0.70	3.5	Vietnam [43]
	Pond	1.9 ± 0.29	1.6 ± 0.16	1.3 ± 0.45	1.4 ± 0.35	0.6	Canadia [44]
Pb	River	220 ± 44	224 ± 36	191 ± 47	239 ± 68	91.3	Vietnam [43]
	Pond	131 ± 24	95 ± 6.8	76 ± 7.2	98 ± 12	35.0	Canadia [44]
Cu	River	118 ± 13	72 ± 7.9	66 ± 9.2	76 ± 6.5	197	Vietnam [43]
	Pond	74 ± 7.1	67 ± 7.4	72 ± 14	64 ± 14	35.7	Canadia [44]
Zn	River	620 ± 89	453 ± 58	332 ± 74	337 ± 71	315	Vietnam [43]
	Pond	324 ± 66	272 ± 22	216 ± 11	248 ± 13	123	Canadia [44]

^i^ Standards for the protection of aquatic life are used in Vietnam [44] and Canada [45].

**Table 2:** Levels of metals in fish muscles in the present study and the maximum acceptable limits (mg/kg ww). The mean values from all sites and the standard error of the mean are shown in parentheses. The values in bold are those that exceed the maximum permissible limits.

Metals	Concentration (Present Study)			^ii^ Maximum Permissible Limits	
	Silver Carp	Common Carp	Tilapia		
Cd	0.002–0.098 (0.023 ± 0.008)	0.003–0.058 (0.024 ± 0.005)	0.001–0.080 (0.015 ± 0.005)	0.05	(46/2007/QĐ-BYT)
				0.05	EC (No.1881/2006)
				1.00	(WHO 1989)
Pb	0.06–1.46 (0.36 ± 0.10)	0.06–1.58 (0.33 ± 0.09)	0.11–1.13 (0.30 ± 0.06)	0.20	(46/2007/QĐ-BYT)
				0.30	EC (No. 1881/2006)
				2.00	(WHO 1989)
Cu	0.5–2.7 (1.03 ± 0.17)	0.5–3.0 (1.53 ± 0.18)	0.5–3.1 (1.27 ± 0.16)	30	(46/2007/QĐ-BYT)
				30	(WHO 1989)
Zn	6.0–16 (11 ± 0.8)	11–54 (10 ± 2.5)	9–42 (16 ± 2.2)	100	(46/2007/QĐ-BYT)
				100	(WHO 1989)

^ii^ Maximum permissible limits for some metals in fish muscle employed in Vietnam [52]; the WHO [53] and EC’s [42] recommended maximum level allowed in the muscle of fish.

**Table 3:** Calculated values for the new classification of the expanded, modified potential ecological risk index (emRI) vs. the potential ecological risk index (RI).

Category	Ecological Risk Grade	Ecological Risk Thresholds	
		(RI; [33])	(emRI; Present Study)
1	Low ecological risk	RI < 150	emRI < 1.1
2	Moderate ecological risk	150 ≤ RI < 300	1.1 ≤ emRI < 2.3
3	Considerable ecological risk	300 ≤ RI < 600	2.3 ≤ emRI < 4.5
4	Very high ecological risk	RI ≥ 600	emRI ≥ 4.5
Sum of toxic units (Hg, Cd, As, Pb, Cu, Cr, Zn, and PCB) [33]		133	

**Table 4:** Modified potential ecological risk factors (mEr) and expanded, modified potential ecological risk indexes (emRI) for metals mixtures in surface sediments of rivers and ponds at different sites.

		mE_r_ (River)				emRI (river)	mE_r_ (Pond)				emRI (Pond)
		Zn	Cu	Pb	Cd		Zn	Cu	Pb	Cd	
Site 1	Spring	10.2	17.4	77	1756	38	8.7	14.0	59.3	760	20.5
	Autumn	7.2	24.7	27	991	26	3.7	22.1	28.2	751	19.6
Average Site 1		8.1 ± 1.3	24 ± 2.4	58 ± 12	1262 ± 324	32	5.7 ± 1.2	19 ± 3.1	41 ± 7.5	755 ± 141	20.0
Site 2	Spring	8.1	10.3	76	1292	34	4.8	10.7	23.0	432	11.5
	Autumn	3.8	15.7	28	1270	28	3.6	18.1	24.6	642	16.8
Average Site 2		6.2 ± 0.8	13 ± 1.6	52 ± 8.7	1282 ± 311	31	4.2 ± 0.5	14 ± 2.0	24 ± 4.0	537 ± 67	14.1
Site 3	Spring	8.2	10.6	86	1534	40	1.8	6.4	10.7	123	3.8
	Autumn	2.7	19.4	25	649	17	1.9	12.1	13.2	385	15.1
Average Site 3		5.4 ± 1.3	15 ± 2.1	55 ± 14	1091 ± 205	28	1.8 ± 0.6	8.8 ± 3.4	12 ± 4.1	235 ± 113	9.4
Site 4	Spring	7.2	16.3	102	1219	33	2.8	6.7	17.3	220	6.0
	Autumn	2.3	16.6	23	569	15	2.9	16.4	23.9	576	15.1
Average Site 4		5.2 ± 1.3	16 ± 2.3	71 ± 25	959 ± 306	26	2.8 ± 0.2	11 ± 2.4	20 ± 2.1	363 ± 100	9.7

**Table 5:** Estimated daily intake (means ± SE) regarding the consumption of different metals in different fishes (μg/kg bw/day). Min and max EDI values are in the parentheses. Estimated weekly intakes (EWI; μg/kg bw/week) for Zn, Cu and Estimated monthly intakes (EMI; μg/kg bw/month) for Cd and acceptable daily intakes (ADI; μg/kg bw/day). Provisional tolerable daily intake (PTWI, µg/week/capita) and provisional tolerable monthly intake (PTMI, µg/month/capita) were set by WHO/FAO [36], in which Zn: 7000, Cu: 3500 µg/kg bw/week, Cd: 25 µg/kg bw/month. Percentage of EWI or EMI in comparison with PTWI or PTMI were presented below each value. * indicates the estimated monthly intakes calculated for Cd.

		EDI				EWI/EMI *			
		Zn	Cu	Cd	Pb	Zn	Cu	Cd *	Pb
Site 1	C. Carp	20.9 ± 4	1.48 ± 0.013	1.9 × 10^−2^ ± 0.19	0.27 ± 0.04	146	10.3	0.56	1.9
		(5.5–70.6)	(0.3–3.0)	(0–0.06)	(0.08–0.66)	2.1%	0.3%	2.3%	
	Tilapia	17 ± 3.2	1.0 ± 0.011	1.2 × 10^−2^ ± 0.14	0.23 ± 0.05	119	7.1	0.36	1.6
		(4.3–54.1)	(0.26–2.6)	(0–0.04)	(0.02–0.72)	1.7%	0.2%	1.4%	
Site 2	C. Carp	14 ± 2.2	0.97 ± 0.007	1.3 × 10^−2^ ± 0.14	0.15 ± 0.02	100	6.8	0.38	1.1
		(4.7–27)	(0.36–1.8)	(0–0.032)	(0.04–0.27)	1.4%	0.2%	1.5%	
	S. Carp	11 ± 2.4	0.53 ± 0.008	7.4 × 10^−3^ ± 0.08	0.16 ± 0.05	75	3.7	0.22	1.1
		(4.6–27.3)	(0.29–0.98)	(0–0.017)	(0.04–0.37)	1.1%	0.11%	0.89%	
	Tilapia	12 ± 2.0	1.0 ± 0.007	1.2 × 10^−2^ ± 0.15	0.24 ± 0.07	85	7.1	0.36	1.7
		(4.1–45)	(0.23–2.61)	(0–0.048)	(0.03–1.1)	1.2%	0.20%	1.4%	
Site 3	C. Carp	15 ± 2.8	1.1 ± 0.009	1.9 × 10^−2^ ± 0.22	0.18 ± 0.04	102	7.7	0.57	1.2
		(7.8–38)	(0.4–2.3)	(0–0.054)	(0.02–0.44)	1.5%	0.20%	2.3%	
	Tilapia	9.6 ± 0.9	1.0 ± 0.003	7.6 × 10^−3^ ± 0.19	0.14 ± 0.02	67	7.0	0.23	1.0
		(6.1–15)	(0.3–2.2)	(0–0.024)	(0.05–0.25)	0.96%	0.20%	0.91%	
Site 4	C. Carp	14 ± 1.7	1.4 ± 0.006	2.0 × 10^−2^ ± 0.24	0.22 ± 0.06	97	9.8	0.59	1.5
		(6.9–26)	(0.26–3.04)	(0–0.072)	(0.04–0.96)	1.4%	0.28%	2.4%	
	S. Carp	9.8 ± 0.9	1.2 ± 0.003	5.7 × 10^−3^ ± 0.23	0.17 ± 0.03	68	8.1	0.17	1.2
		(6.0–16)	(0.28–2.64)	(0–0.024)	(0.02–0.39)	0.98%	0.23%	0.68%	
	Tilapia	9.6 ± 1.1	1.1 ± 0.004	7.8 × 10^−3^ ± 0.29	0.18 ± 0.04	67	7.5	0.24	1.3
		(5.1–16)	(0.32–2.9)	(0–0.032)	(0.02–0.47)	0.96%	0.22%	0.94%	
ADI		1000	500	0.83					
PTWI or PTMI *						7000	3500	25 *	

## Data Availability

Data sharing is not applicable to this article.
